# Linear and non linear measures of pupil size as a function of hypnotizability

**DOI:** 10.1038/s41598-021-84756-y

**Published:** 2021-03-04

**Authors:** Antonio Lanatà, Alberto Greco, Mirco Ciardelli, Allison Uvelli, Elisabetta Fratini, Diego Manzoni, Enzo P. Scilingo, Enrica L. Santarcangelo, Laura Sebastiani

**Affiliations:** 1 Department of Information Engineering, University of Florence, Florence, Italy; 2 Research Center “E. Piaggio”, University of Pisa, Pisa, Italy; 3 Department of Translational Research and New Technologies in Medicine and Surgery, University of Pisa, Via San Zeno, 31, 56127 Pisa, Italy; 4 Department of Information Engineering, University of Pisa, Pisa, Italy

**Keywords:** Neuroscience, Cognitive neuroscience, Attention, Cognitive control, Consciousness

## Abstract

Higher arousal and cortical excitability have been observed in high hypnotizable individuals (highs) with respect to low hypnotizables (lows), which may be due to differences in the activation of ascending activating systems. The present study investigated the possible hypnotizability-related difference in the cortical noradrenergic tone sustained by the activity of the Locus Coeruleus which is strongly related to pupil size. This was measured during relaxation in three groups of participants—highs (N = 15), lows (N = 15) and medium hypnotizable individuals (mediums, N = 11)—in the time and frequency domains and through the Recurrence Quantification Analysis. ECG and Skin Conductace (SC) were monitored to extract autonomic indices of relaxation (heart interbeats intervals, parasympathetic component of heart rate variability (RMSSD) and tonic SC (MeanTonicSC). Most variables indicated that participants relaxed throughout the session. Pupil features did not show significant differences between highs, mediums and lows, except for the spectral Band Median Frequency which was higher in mediums than in lows and highs at the beginning, but not at the end of the session.Thus, the present findings of pupil size cannot account for the differences in arousal and motor cortex excitability observed between highs and lows in resting conditions.

## Introduction

Hypnotizability is a well-known individual trait owing to the ability of the persons scoring high on hypnotizability scales (highs) to modify perception, memory and behaviour according to specific imaginative instructions named “suggestions”^1^ and, in particular, to control pain through cognitive strategies^2^. Hypnotizability has a gaussian distribution in the general population^3^ and is associated with several cognitive-emotional^4–10^, sensorimotor and cardiovascular features^11,12^ which are observable also in the ordinary state of consciousness and in the absence of suggestions. Hypnotizability-related brain morpho-functional differences have been described in the Salience, Executive, Default Mode circuits^13^ and in the cerebellum^14^. In particular, fMRI revealed stronger functional connectivity between the dorsolateral prefrontal cortex and the anterior cingulate cortex, which has been advocated as responsible for part of the hypnotic phenomenology^15^, together with the highs’ strong functional equivalence between imagery and perception suggested by behavioural research and confirmed by EEG studies^16^**.** The latter finding—stronger functional equivalence between imagery and perception in highs than in lows—suggested greater excitability of the motor cortex in highs with respect to lows. During both resting and motor imagery conditions, indeed, Transcranial Magnetic Stimulation of the motor cortex showed greater cortical excitability in highs than in low hypnotizables (lows), with medium hypnotizable individuals (mediums)—who better represent the general population^3^—exhibiting intermediate values^17^.

Higher arousal was also suggested in highs by research conducted through the Attention Network Test which showed higher efficiency in highs than in lows in achieving and maintaining their readiness to respond to incoming stimuli as measured by reaction times^18^. Moreover, encoding of verbal priming^19^, emotional face recognition^20^ and the speed of visual processing^21^ were associated with shorter reaction times in highs than in lows.

Such differences—the highs’ higher arousal and excitability of the motor cortex- could be sustained by higher tonic activity of ascending activating systems^22^. In fact, higher dopaminergic tone has been hypothesized for highs^5^ based on their greater attentional stability^23,24^, on their reduced dopamine catabolism—suggested, although not unanimously, by studies of Cathechol-O-Methil Transferase polymorphism^25–29^—and on the larger content of homovanillic acid measured in their cerebrospinal fluid^30^. On the other hand, noradrenaline shares its degradation pattern with dopamine, thus both neurotransmitters could be responsible for the amount of homovanillic acid found in the cerebrospinal fluid. Thus, the highs’ higher cortical excitability and arousal^18–20,31,32^ could be due also to higher noradrenergic tone, although no information about the possible contribution of nor-adrenergic and colinergic pathways are currently available**.**

The Locus Coeruleus (LC), located in the upper dorsolateral pontine tegmentum, is the primary source of noradrenergic supply to the cortex^33–36^. It diffusely projects to several cortical and subcortical regions including the primary motor, orbitofrontal, medial prefrontal and anterior cingulate cortices, the periaqueductal grey matter and preganglionic sympathetic and parasympathetic nuclei^37–40^. LC controls pupil dilation by inhibiting the preganglionic parasympathetic neurons located in the nucleus of Edinger-Westphal^41–44^, which innervates the iris sphincter muscle responsible for pupil dilation^36,45^. Although also the release of acetylcholine from the basal forebrain contributes to regulate the pupil size spontaneous oscillations^45–48^, in animals the activity of noradrenergic axons closely follows the spontaneous fluctuations in pupil dilation during rest and and motor tasks^47^ and, in humans, the pupil diameter is positively correlated with the activity of the rostral LC during rest and attentional tasks^49^. Thus**,** pupil size is considered a reliable index of LC noradrenergic activity^36,47,50^. In particular, the relationship between the spontaneous changes in tonic pupil size and the cognitive state follows an inverted U-shape^51^, and pupil dilation increases during several cognitive tasks with the mydriasis positively associated with the tasks demand, i.e. high mental load/conflict^51–57^.

In resting conditions, there is a common sympathetic control of pupil size and skin conductance and common parasympathetic and sympathetic controls of pupil size and heart rate^58^. Thus, we may expect a modulation of heart rate and skin conductance congruent with the changes in pupil size.

The present study aimed at investigating whether, at the beginning of a relaxation session, highs exhibit greater LC related noradrenergic tone with respect to lows by evaluating pupil features in highs (N = 15, 8 females), lows (N = 15, 10 females) and mediums (N = 11, 6 females) who often exhibit psychophysiological characteristics intermediate between highs’and lows^2^ and sometimes are similar to one of the two groups^9^. We have to remark, however, that, in contrast to recent recommendations^59^, most of current evidence compared only highs and lows.

During the experimental session, participants were invited to relax at their best without any further instruction^60^ three times (trials: T1, T2, T3 lasting 6 min each) separated from each other by a 2 min interval of plane conversation with one of the experimenters.ECG and Skin Conductace (SC) were monitored to extract indices of relaxation (heart interbeats intervals (RR), parasympathetic component of heart rate variability (RMSSD) and tonic SC (MeanTonicSC). During the entire session, particiants had to fixate the cross appearing at the central point of the screen. During relaxation, parasympathetic prevalence^60,61^ and lower activity of LC should induce (1) decrease in pupil size, (2) increase in heart inter-beat interval duration (Mean RR) and in the parasympathetically-mediated variability of RR (represented in the time domain by the Root Mean Square of Successive RR differences, RMSSD), (3) decrease in Mean Tonic Skin Conductance^58,62^. Variables were studied in the earliest (I_1_) and latest minute (I_6_) of T1, T2, T3.

Both linear and non linear methods of analysis were used. The former allow the comparison of findings with earlier reports, although the latter seems to be more appropriate to the analysis of complex systems like biological phenomena^63^.

## Results

State anxiety scores (STAI-X ), collected immediately before the experimental session in order to exclude groups differences possibly biasing psychophysiological measures, were within the normality range^64^. A few participants, however, experienced mild anxiety compatible with the laboratory condition (STAI scores: 40–50 = mild anxiety; 50–60 = moderate anxiety; > 60 = severe anxiety). One-way ANOVA did not reveal significant differences between hypnotizability groups (mean ± SD; highs, 43.61 ± 10.61; mediums, 43.50 ± 9.46; lows, 41.15 ± 8.62).

### Heart rate, heart rate parasympathetic variability and skin conductance indicate relaxation

**Figure 1:**
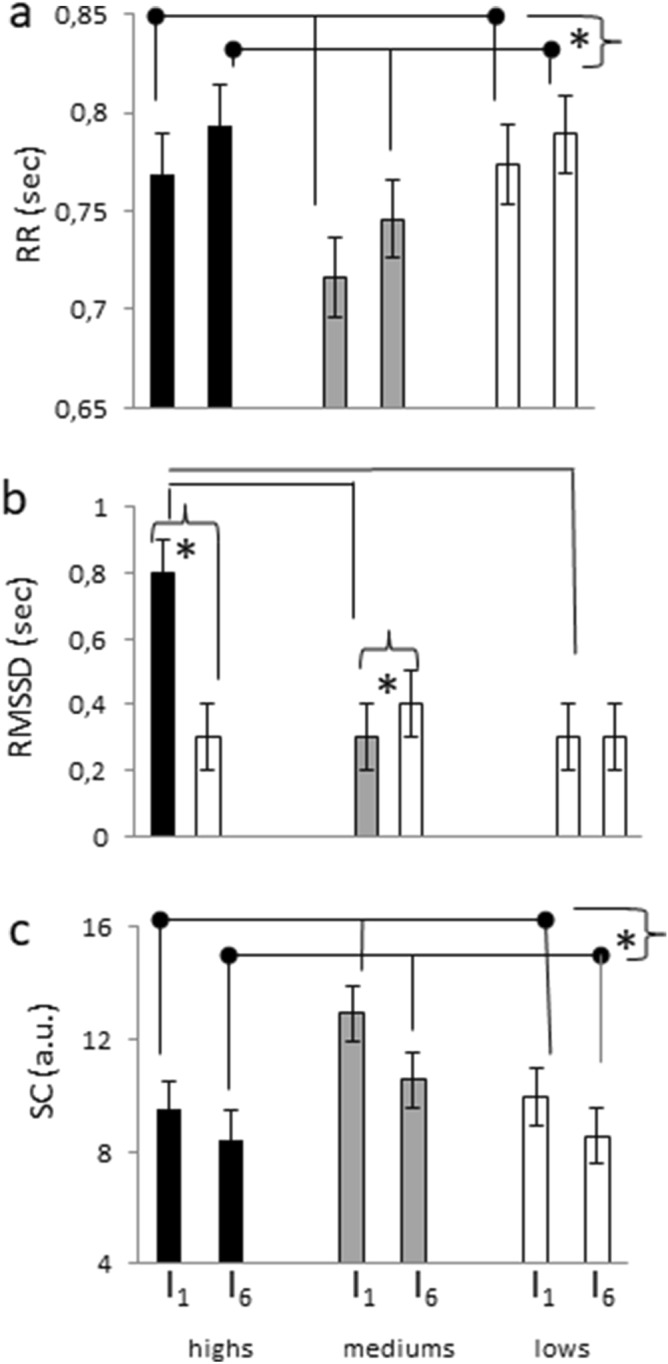
Autonomic variables (mean, SEM). (**a**) Mean RR (interbeat interval, sec; (**b**) Root Mean Square of Successive Differences (RMSSD, sec); (**c**) mean Tonic Skin Conductance (SC, arbitrary units). I_1_ and I_6_: first and last Interval. Stars indicate significant differences between Intervals independently from hypnotizability for RR and SC (a: I_1_ < I_6_; c: I_1_ > I_6_) and (**b**) between Intervals in highs and mediums for RMSSD. Lines (**b**) indicate significant hypnotizability differences in I_1_.

The changes in mean heart interbeats interval (mean RR) and MeanTonic Skin Conductance (Mean SC) from the earliest (I_1_) to the latest interval (I_6_) of relaxation indicated that all groups of participants relaxed. Repeated measures ANOVA, in fact, yielded a significant Interval effect (F(1,38) = 6.893, *p* = 0.012, η^2^ = 0.126) with MeanRR (Fig. 1a) significantly longer in I_6_ (mean ± SD (sec); 0.779 ± 0.140) than in I_1_ (0.756 ± 0.137).

The Root Mean Square of Successive RR Differences between normal heartbeats (RMSSD) differed between groups (Kruskal Wallis test) in the earliest interval of relaxation (I_1_, *p* = 0.00006) but not in the latest interval (I_6_, *p* = 0.310) independently from Trials. Its value was higher in highs than in mediums (*p* = 0.00011) and lows (*p* = 0.00013), with no significant difference between mediums and lows (Fig. 1b). RMSSD increased from I_1_ to I_6_ (Wilcoxon test) in mediums (*p* = 0.0012), decreased in highs (*p* = 0.001), and did not change in lows (*p* = 0.534).

Repeated measure ANOVA applied to MeanTonic SC revealed a significant Interval effect (F(1,51) = 32.321, *p* = 0.00013, η^2^ = 0.388) with higher values in I_1_ than in I_6_ (Fig. 1c) in all groups.

### Time domain linear measures do not reveal hynotizability-related differences in pupil size

**Figure 2:**
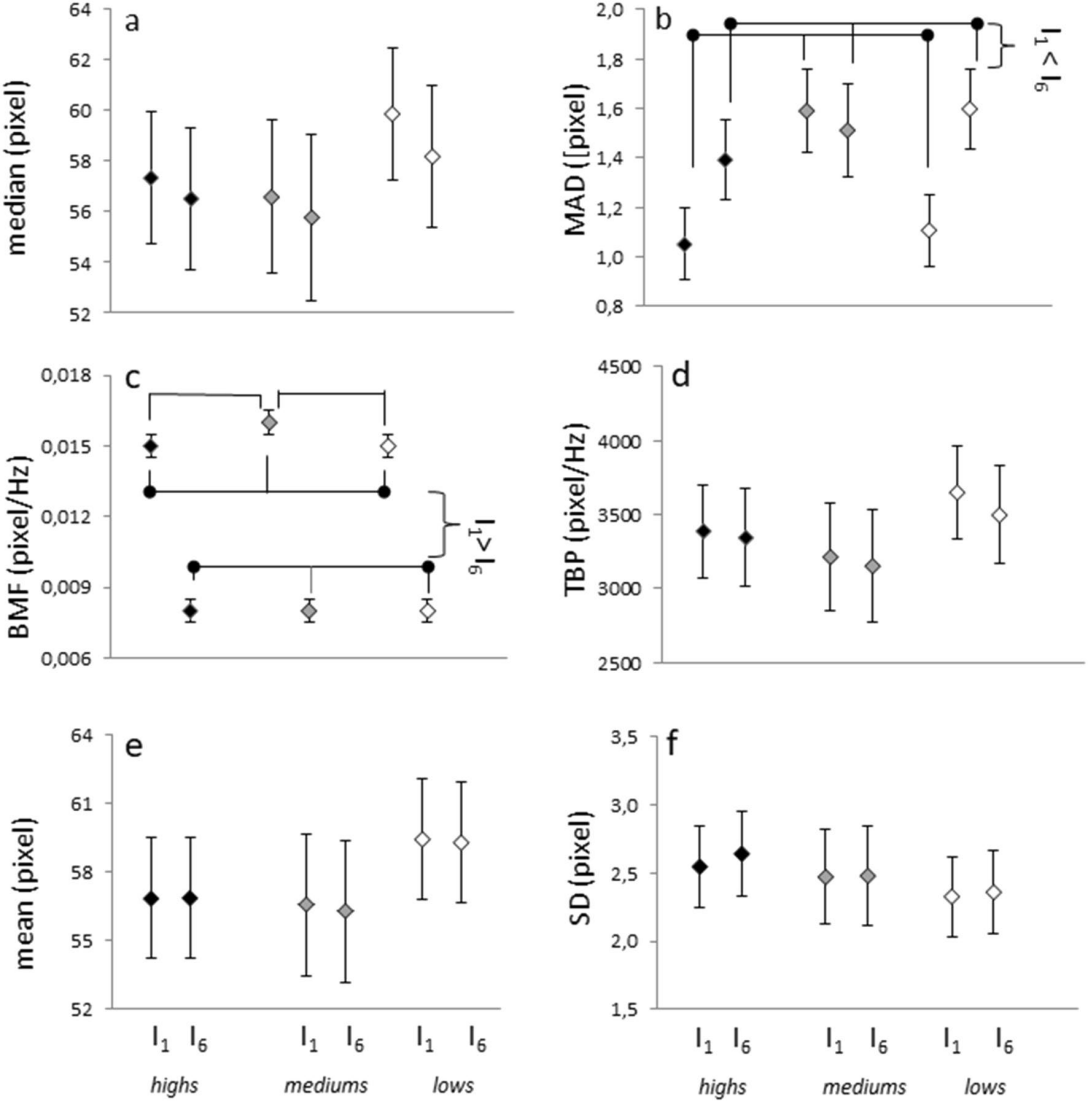
Pupil diameter features (mean, SEM). (**a**) median diameter; (**b**) MAD, the median value of the absolute deviations from the median value; (**c**) BMF, median band frequency; (**d**) TBP, band total power. Lines indicate significant Interval effects for MAD and the MBF independently from hypnotizability. (**e, f**): mean pupil size and SD.

In the time domain, repeated measures ANOVA did not reveal significant differences between hypnotizability groups for the median value of the pupil diameter (Me), which did not change significantly during relaxation (Fig. 2a), and for the pupil size median variability (MAD) (Fig. 2b), which exhibited a significant Interval effect indicating an increase at the end of relaxation independently from hypnotizability (I_1_ < I_6_, F(2,38) = 6.995, *p* = 0.012, η^2^ = 0.155).

No significant result would have been obtained by using mean values (Mean) and standard deviations (SD) instead of Me and MAD. Mean values (Fig. 2e), in fact, exhibited a significant Trial x Interval interaction (F(2,38) = 3.961, *p* = 0.024, η^2^ = 0.094) not surviving to Bonferroni correction and sustained by a significant difference between intervals only in the third trial (I_1_ < I_6_, t = 2.661, *p* = 0.011). A Trial effect not surviving to Bonferroni correction (Fig. 2f) was found for SD (F(2,76) = 3.620, *p* = 0.033, η^2^ = 0.087) with a significant difference only between T1 and T2 (F(2,38) = 6.054, *p* = 0.019). They are here reported to allow the comparison with other authors’ findings.

### Frequency domain linear measures reveal hypnotizability related differences in pupil size

In the frequency domain, the Band Median Frequency (BMF), indicates the frequency band dividing the pupil size power spectrum into two regions with equal amplitude. Nonparametric statistics revealed a significant difference between hypnotizability groups for BMF in I_1_ (*p* = 0.000008) but not in I_6_ (Fig. 2c). In I_1_ a significant difference was observed between lows and mediums (*p* = 0.0000185) and between highs and mediums (*p* = 0.000019). All groups decreased their BMF from I_1_ to I_6_ (highs, *p* = 0.00065; lows, *p* = 0.00065; mediums, *p* = 0.0033).

**Figure 3:**
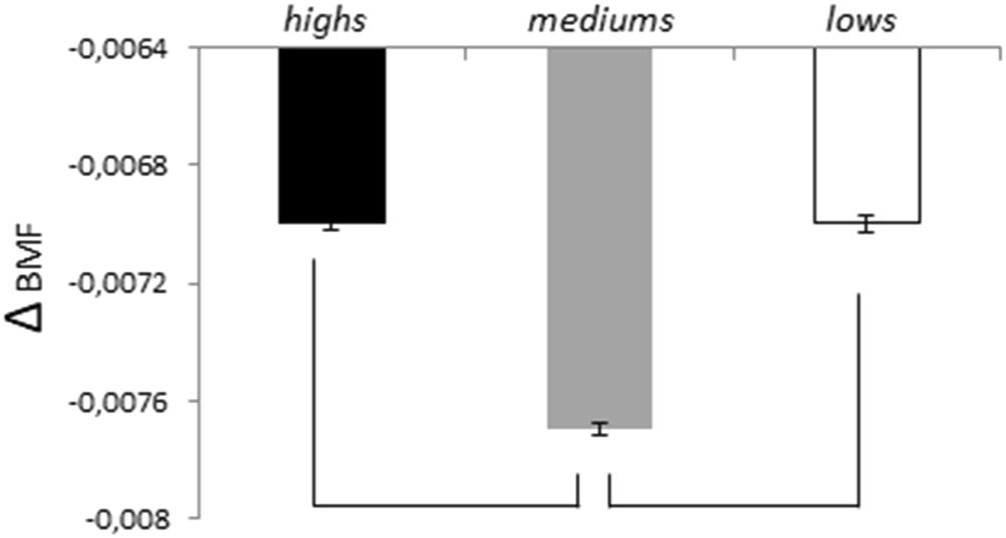
Changes (Δ_I6-I1_) in the pupil spectrum Band Median Frequency. BMF (Mean, SEM). Lines indicate significant differences between mediums and highs/lows.

We also compared the BMF changes observed in the three groups from I_1_ to I_6_ (Fig. 3) by Kruskal Wallis test applied to the difference between I_6_ and I_1_ (Δ_(I6-I1)_) and observed a highly significant Hypnotizability effect (*p* = 0.0000072) with the mediums’decrease larger than both highs’(*p* = 0.000019) and lows’(*p* = 0.000019).

ANOVA did not reveal significant effects and interactions for the Total Band Power (TBP), which indicates the pupil spectrum total power (Fig. 2d).

### The Recurrence Plot shows a trend toward hypnotizability-related differences in pupil size

**Figure 4:**
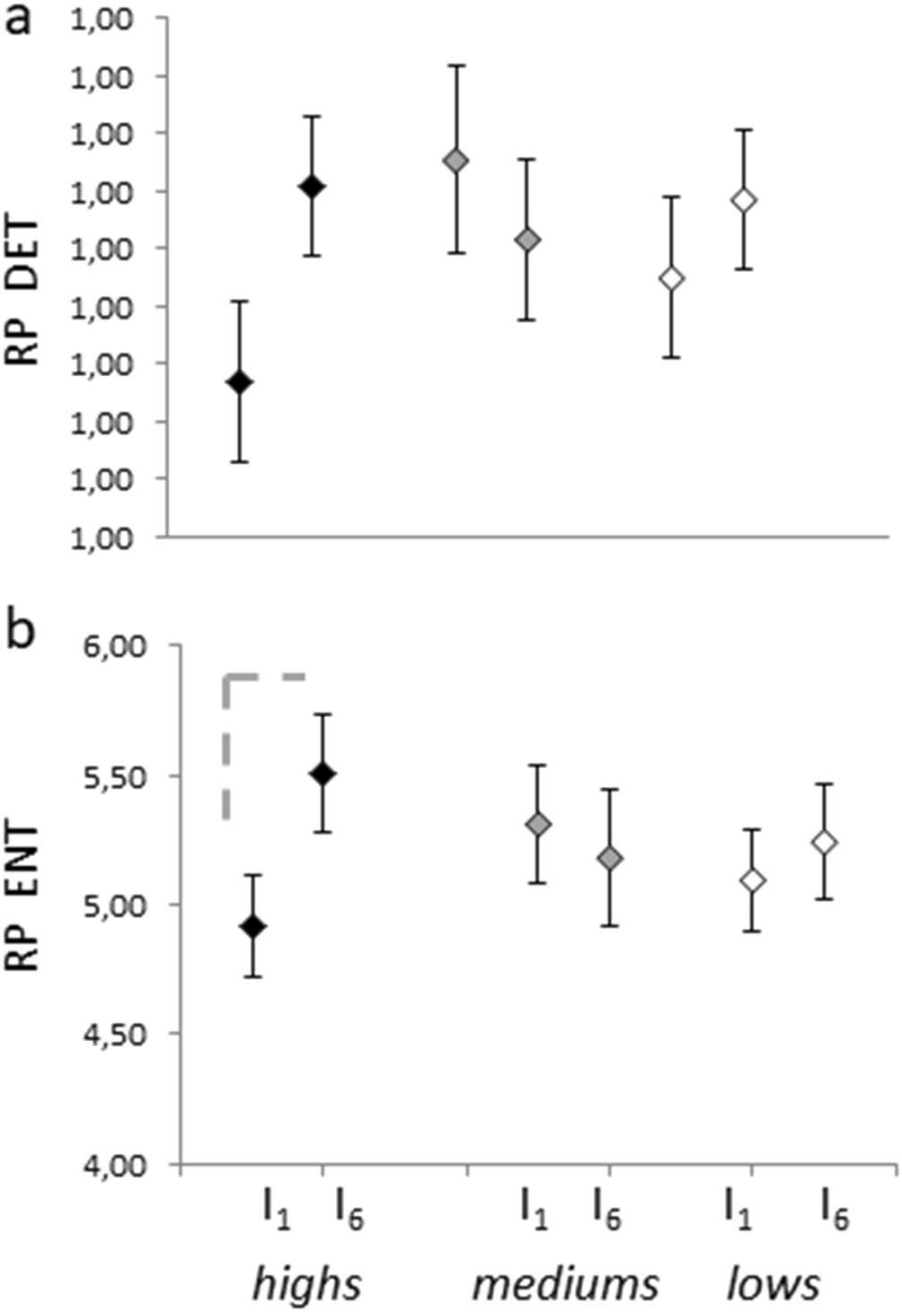
Determinism and Entropy of the pupil signal Recurrence Plot. (**a**) Determinism and (**b**), Entropy in I_1_ and I_6_. The dotted line indicates differences approaching significance (*p* = .029 with the level of significance set at *p* = 0.025 after Bonferroni correction).

Determinism extracted from the pupil size Recurrence Plot was not different between highs, mediums and lows and did not change from I_1_ to I_6_ (Fig. 4a). In contrast, the Interval x Hypnotizability interaction observed for Entropy (Fig. 4b) approached significance (F(2,38) = 3.907, *p* = 0.029, η^2^ = 0.171), with a significant increase from I_1_ to I_6_ in highs (F(1,14) = 6.909, *p* = 0.02), but not in mediums and lows.

## Discussion

The main aim of the study was to assess whether the highs’ higher arousal level^18–21,31^ and greater cortical excitability^17^ with respect to lows could be sustained by greater noradrenergic tone due to LC activity. Findings show that this was not the case, as there was no significant difference in the median pupil size and variability between hypnotizability groups. The similar pupil’s features in highs and lows, however, do not exclude a different noradrenergic supply to the cortex by other structures, e.g. brainstem nuclei^36^. Thus, present findings, in fact, exclude only the LC noradrenergic contribution to the difference in the cortical excitability observed between highs and lows^17^ and to their different arousal level^18–21^. Similar pupil diameters in highs, mediums and lows at the end of a relaxation task, however, do not imply similar responses to tasks involving the LC phasic activity.

The findings concerning MAD and BMF, which increases and decreases, respectively, from the beginning to the end of relaxation, could reflect an attempt of participants to remain alert during the long lasting task to fixate a point inducing sympathetic activation^65^. The pupil diameter spontaneous oscillation, which can be described by the diameter power spectrum, shows higher BMF values in mediums than in highs and lows in I_1_. This indicates that at the beginning of the relaxation session (I_1_) the mediums’ pupil spectrum frequencies were slighly displaced toward parasympathetic values and is in line with the greater values of RMSSD observed in mediums. Their larger decrease in BMF (Δ_I6-I1_)with respect to highs’and lows’could be due to greater engagement in the attempt to relax or to cognitive fatigue and indicates a larger shift toward a sympathetic control possibly related to the cognitive cost of the relaxation task^66^. BMF, in fact, has been proposed as an index of the changes occurring in the cortical state owing to the correlation betwen pupil size and the activity of noradrenergic neurons of LC and of its network linked to arousal, attention and perception systems^67^.

The sensitivity of the pupil signal to hypnotizability has been indicated also by non linear analysis. In fact, the Hypnotizability x Interval interaction observed for Entropy and approaching significance indicates an increase in the pupil signal complexity across relaxation only in highs. This contrasts with the significant increase in the complexity of the pupil signal observed during relaxation in the general population^67^ which includes highs, mediums and lows. The inconsistent results could be accounted for by differences in the administered instructions of relaxation, as well as by different experimental conditions. An intriguing observation is that, in contrast to the highs’ increase in the complexity of the pupil signal, earlier studies of the EEG signal during relaxation revealed an increase in the RP Determinism at centroparietal level and no change in the RP Entropy^68^. Since we would expect similar behavior for pupil and brain, as the LC noradrenergic activity is widely distributed to the cerebral cortex, further studies will address this point.

The changes in MeanRR and MeanTonic SC occurring from the first to the latest interval indicate that during the experimental session all groups relaxed*,* although through different mechanisms. In fact, highs decreased their RMSSD, likely to greater involvement in the cognitive task of relaxation^66^, mediums increased and lows did not change it during relaxation. Thus, similar pupil features and changes were observed in groups with different autonomic balance. Our findings are not entirely consistent with the view that, in resting conditions, heart rate and pupil size are under a similar autonomic control^58^. In fact, in the present study, the MeanRR increase and MeanTonic SC decrease indicate a shift toward a parasympathetic prevalence^61,62^, whereas the pupil size median variability increase and the spectral median frequency decrease suggest a shift toward sympathetic control^65,69^. We may hypothesize, in this respect, that the afferences conveying muscles and interoceptive signals directly to cardiorespiratory centers could be more effective that the LC control on these centers. In contrast, LC activity could be more sensitive to cognitive activities, which might, thus, induce changes in pupil size partially independent from the bodily state.Evidence of dissociation between mental fatigue and cardiac activity, in fact, has been reported^70^. A limitation of the study, in fact, is that the participants’ cognitive-emotional processes occurring during relaxation were not controlled. Manipulation of working memory, planning, mind-wandering, mental imagery, would increase tonic pupil size and our findings may have been biased by not reported cognitive activities^52–55,71^. Moreover, personality and intelligence^72^ are known to modulate pupil diameter. In this respect, although participants were all young, healthy volunteers attending the University of Pisa, we cannot exclude that these factor possibly biased our results*.* Another limitation is related to the several neurotransmitters—vasopressin, somatostatin, neuropeptide Y, enkephalin, neurotensin, corticotropin-releasing factor, galanin—which control LC^73,74^ and could induce a large variability in its activity according to bodily and environmental conditions, thus limiting the generalization of present findings. Another limitation of the study is that relaxation was assessed on the basis of autonomic variables but not supported by self-reports which were not collected. We can exclude, however, biasing factor related to luminance and environmental stimuli because they were strictly controlled.

In conclusion, findings indicate that before relaxation -which is a cognitive task performed in highs and lows differentially^66^—the pupil tonic sympathetic control measured by the Band Median Frequency of the pupil diameter power spectrum has a U-shaped relation with hypnotizability. Thus, the LC dependent noradrenergic tone cannot account for the differences observed between highs and lows in cortical excitability and arousal^17–21^. It may be interesting to notice that the observed hypnotizability-related difference in pupil BMF disappears at the end of relaxation, similarly to what occurs for the Determinism of the EEG Recurrence Plot^68^, so that we can hypothesize that hypnotizability-related basal psychophysiological differences may be buffered through the participants’ own cognitive strategies. Finally, linear analysis in the frequency domain and non linear analysis seem to be more sensitive than linear methods in the time domain to the hypnotizability-related modulation of the pupil signal.

## Methods

### Subjects

The experimental protocol was approved by the Bioethics Committee of the University of Pisa (n. 3/2019. Forthy-one healthy volunteers of both gender (age, 20–24 years) signed an informed consent and were enrolled in the study, which was conducted according to the Declaration of Helsinki. Exclusion criteria were the anamnesis of medical, neurological and psychiatric disorders, attention deficits, psychoactive drugs intake in the latest 3 months. Since hypnotizability is a substantially stable trait^75^, participants were selected among those who had been submitted to hypnotic assessment through the Italian version of the Stanford Scale of Hypnotic Susceptibility (SHSS, score 0–12), form A^76^ between 8 and 12 months before receiving the telephone call inviting them to participate in the present study. Among the consecutive volunteers who accepted to be enrolled, 15 were highly hypnotizable (highs, SHSS score ≥ 8, 10 females), 11 were medium hypnotizable (mediums, SHSS score between 5 and 7, 6 females) and 15 were low hypnotizable (lows, SHSS score < 4, 10 females). Data have been uploaded as Supplementary Electronic Material.

### Experimental procedure

Before the experimental session, participants completed the State Anxiety Inventory (STAI-X)^64^ to ascertain the absence of basal differences in anxiety which could bias the pupil response to the procedure^77^. Then, they sat in a comfortable armchair in a sound attenuated room with constant temperature (20°) and luminance (neon light illumination) and were invited to relax at their best and to avoid the contact between teeth arches to prevent trigeminal stimulation possibly leading to LC activation^78^. ECG, Skin Conductance (SC) and the right pupil diameter were recorded during a simple relaxation session^60^ lasting 6 min, after 5 min of familiarization with the experimental setting**.** Experimenters were in the same room but not visible to participants. Movements were not allowed during the session. The participants’ eye level was adjusted by modifying the armchair height from the flow to allow them to look at the screen easily and each subject was asked to fixate at the highlighted central point of the screen.

ECG and SC were acquired using the BIOPAC MP35 system (Biopac Systems Inc., CA, USA) with a sampling frequency of 500 Hz.

**Figure 5:**
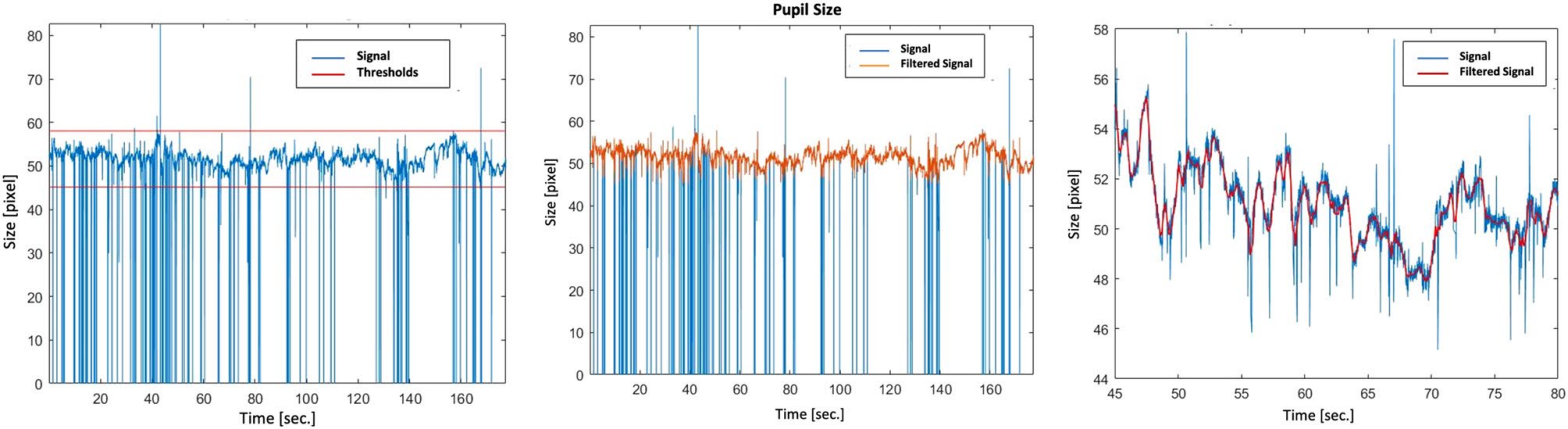
Processing of the pupil signal. In the first graph, thresholds are in red; in the second graph signal after threshold is in orange; in the third graph averaged mean filtered signal is in red; in all graphs raw signal is in blue.

A Pupil Labs Eye-tracking open source platform (https://pupil-labs.com) supplied with Software Pupil Labs (Pupil Capture, Player) and with Microsoft Visual Studio software prepared ad hoc was used to monitor the eyes’ physiological information and the subjects’ point of gaze. Specifically, a single lens system was adopted to perform pupillometry and gaze point measure at a sampling frequency of 100 Hz. Only pupil diameter was retained for further analysis^79^. Before starting the experiments, a calibration process has been performed to map each eye position acquired by the camera to the correspondent coordinates on the screen (i.e., the calibration is included in the Pupil Labs acquisition software). All artifacts due to blinks and saccades have been coorected through a two steps process. First two thresholds (*up* and *down*) have been applied to detec all outliers, then an average mean filter has applied to smooth the signal. Specifically, the thresholds have been computed as follows (Fig. 5):

$$thr_{up} = median\left( x \right) + 5 mad\left( x \right);\quad thr_{down} = median\left( x \right) - 5\; mad\left( x \right);$$

Furthermore, subject pupil data were normalized on the pupil data distribution over the basal session according to the following formula:

$$x^{\prime} = \frac{{x - 5th\; percentie\left( {x_{basal} } \right)}}{{95th\; percentile\left( {x_{basale} } \right) - 5th\; percentile\left( {x_{basal} } \right)}}$$

This normalization enables robust data scaling to outliers and groups' comparison.

### Variables

Pupil Linear variables. Pupil features were extracted in the time and frequency domain. In the time domain, in order to increase the reliability of findings, we considered pupil features less sensitive to outliers with respect to the classically investigated mean diameter and standard deviation^80^ and, thus, computed (a) the pupil median value (Me) which represents the value lying at the midpoint of an ordered frequency distribution such that there is an equal probability of falling above or below it), and (b) the Median Absolute Deviations from the median value (MAD), which is defined, for a setx_1,x_2,…,x_n, as the median of the absolute value of the deviations of the data from the median, that is MAD = median(|x_i-median(x)|).

In the frequency domain, we computed the Total Band Power spectrum (TBP) and the Band Median Frequency (BMF). TBP represents the total spectral activity of the pupil diameter, which is due to the pupil size fluctuation. Since the larger the pupil, the lower the fluctuation amplitude^36^, we may expect that larger pupils will be associated with lower TBP, assuming no relevant changes in the frequency bandwidth. BMF indicates the frequency component at which the power spectrum is divided into two regions with equal amplitude. Since pupil size varies with the heart rate variability^62^, we can assume that the pupil signal spectrum contains more high or low spectral frequencies as a function of a pre-eminently parasympathetic and sympathetic control, respectively, in parallel with the RR series spectral content^81^. In fact, a positive correlation has been found between RR and pupil size in resting conditions^58^ and the ratio between low (0–1.6 Hz) and high frequency (1.6–4 Hz) bands (LF/HF ratio) of power spectral densities of the pupillary signal is sensitive to the cognitive load^82^.

TBP was estimated through the periodogram method^83^ applied to the pupillometry time series, with each segment first windowed with a Hamming window. BMF was computed in two steps, first, adding the signal intensity in the whole spectrum and dividing it by 2. In the second step, the median frequency is the first component at which the cumulative frequency exceeds the computed value in step 1^84^.

Pupil nonlinear variables. Recurrence Quantification Analysis has been used to describe several biological systems^85,86^ including the pupil oscillatory behavior^87^ and has been applied also to EEG^68,88^ and heart rate^89^ of subjects with different hypnotizability.

Pupil signal has been used to implement the Recurrence Quantification Analysis (RQA), which is a method for quantifying the dynamic properties of a system represented in the phase space (PS)^90,91^. In our hypothesis, the pupil signal is the outcome of an inner dynamic system. We applied the well-known Takens theorem^92^ to reconstruct its phase space.

The theorem guarantees that the PS geometrical properties of a given nonlinear system can be reconstructed by using copies of the measured times series, as the output of the original system. It is a vector space^93^ in which we can describe the system dynamics by an m-dimensional map, by using a time delay embedding method. In the univariate case, it is represented by the following embedding vector x_n_ = (x_n_,x_n−τ_,…,x_n−(m−1)τ_) where *n* = *1,…,N* is the measured time series, *m* is the embedding dimension, i.e., the number of components in x_n_, and τ is the time delay. In this study, we computed the embedding dimension, *m*, as the first minimum of the false nearest neighbours function over the possible dimensions from zero to ten. An embedding dimension of m = 4 was obtained^94,95^ while the time delay τ was computed as the first minimum of the mutual information profile, maximizing the independence among the components of the embedding vector. Finally, we applied the RQA to quantify the dynamics of the pupil evolution throughout the process. Specifically, RQA is a quantification of the recurrence plot (RP), which is a graph that shows those instants during which a state of the dynamical system recurs, i.e., RP reveals all the time points when the phase space trajectory visits roughly the same area in the phase space. Generally, recurrence points are represented by the following formula:

$$Ri,j = \Theta (\varepsilon - \left| {\left| {xi - xj} \right|} \right|), \quad i,j = 1, \ldots ,N,$$

where N is the number of measured points *x*_*i*_, *ε* is a threshold distance, || ∗|| is a norm, e.g., the Euclidean norm, and Θ(x) is the Heaviside function. Being the value of ε of great importance to have a method able to learn the recurrence structure of the underlying system, we used a customized value of ε for each time series as reported in^96^. In this study, we computed the following features^97^: Determinism (DET) and Entropy (ENTR).

The determinism (DET) is defined as the percentage of recurrence points which form diagonal lines. Let us to consider *x*_*i*_ the time series of one variable, for *m* variables we have *x*_*i*_ = *(x*_*1,i*_*,…,x*_*m,i*_*),* with i = 1,…,N. We define the recurrence matrix, N × N , of element *R*_*ij*_ as follows:

$${\text{Rij}} = \left\{ {\begin{array}{*{20}l} {1,} \hfill & \quad {if\; d\left( {x_{i} ,x_{j} } \right) < \epsilon} \hfill \\ {0,} \hfill & \quad {otherwise} \hfill \\ \end{array} } \right.$$

where *d* is the distance between *x*_*i*_ and *x*_*j*_. The recurrence exits when *R*_*ij*_ = *1* with i ≠ j, the total number of recurrences is $R = \mathop \sum \nolimits_{i = 1}^{N - 1} \mathop \sum \nolimits_{j = i + 1}^{N} R_{ij}$.

Hence,

$$DET = \frac{{\mathop \sum \nolimits_{{l = l_{min}^{D} }}^{N} lP_{D} \left( l \right)}}{{\mathop \sum \nolimits_{l = 1}^{N} lP_{D} \left( l \right)}}$$

where D is defined as the set of diagonal lines; P_D_(l) as the histograms corresponding to the number of lines of the set D with length l > l_min_. DET can be interpreted as the probability that two closely evolving segments of the phase space trajectory will remain close for the next time step. This measure provides indications on the predictability of the dynamical system. Of note, a deterministic process has a recurrence plot with very few single dots but many long diagonal lines, on the contrary, white noise has a recurrence plot with almost only single dots and very few diagonal lines.

The Entropy (ENTR) is defined as the Shannon entropy. Let’s define p(l) as the probability that a diagonal line has exactly length l = l_min_. This can be estimated from the frequency distribution of the probability distribution of the diagonal line lengths:

$$p\left( l \right) = \frac{{P_{D} \left( l \right)}}{{\mathop \sum \nolimits_{{l = l_{min} }}^{D} P_{D} \left( l \right)}}$$

Hence,

$$ENTR = - \mathop \sum \limits_{{l = l_{min} }}^{N} p\left( l \right)\ln p\left( l \right)$$

ENTR refers to the Shannon entropy of the probability p(l) of finding a diagonal line of exactly length l. It reflects the complexity of the RP with respect to the diagonal lines and indicates the complexity of the deterministic structure in the system.

ECG and SC-derived variables. ECG has been analyzed to detect the R-peaks of the QRS-algorithm^98^. Therefore, recorded ECG signals were band-pass filtered (0.05–40 Hz) to reduce noise and movement artefacts. From the detected R-peaks, the RR interval time series were derived. In addition, possible physiological (e.g., ectopic beats) or algorithmic (e.g., R-peak mis-detection) artefacts in RR time series were corrected after visual inspection.

From the RR time series we calculated mean RR interval value (MeanRR) and the square root of the mean squared differences between successive RR intervals (RMSSD) as recommended by the Task Force of the European Society of Cardiology and the North American Society of Pacing and Electrophysiology^81^.

The cvxEDA algorithm was applied to each SC time-series to perform a rigorous and robust decomposition into its tonic and phasic components^99^. Once the tonic signals were estimated the mean value was computed (MeanTonicSC).

### Statistical analysis

The SPSS.15 statistical Package was used for analyses. Since each recording interval lasted 1 min, the earliest and latest interval could provide sufficiently reliable information on the changes occurring from the beginning to the end of the session. Including 6 intervals would have reduced the statistical power without any further advantage.

After normality assessment, non parametric statistics (Kruskal–Wallis test between hypnotizability groups, Wilcoxon test between Intervals) or ANOVA (with Greenhouse–Geisser $\upvarepsilon$ correction when necessary, Hochberg adjustment for multiple comparisons between groups) were applied to STAI scores, MeanRR, RMSSD, MeanTonic SC and to pupil size linear (Median, MAD, BMF, TBP) and nonlinear variables (Recurrence Plot Determinism and Entropy) to assess Hypnotizability (highs, mediums, lows), Trial (T1, T2, T3) and Interval (first interval I_1_, last interval I_6_) depending differences. Bonferroni correction for multiple comparisons was applied and the significance level was set at *p* = 0.013 for the pupil diameter linear variables, *p* = 0.025 for nonlinear variables, *p* = 0.025 for RR and RMSSD, *p* = 0.05 for MeanTonic SC.

## Supplementary Information

Supplementary Information 1.

Supplementary Information 2.

Supplementary Information 3.

Supplementary Information 4.

Supplementary Information 5.

Supplementary Information 6.

Supplementary Information 7.

Supplementary Information 8.

## Data Availability

Data have been uploaded as Supplementary Electronic Material.
