# Supplementary material for: Linear and non linear measures of pupil size as a function of hypnotizability
Source: Sci Rep. 2021 Mar 4;11:5196. doi: 10.1038/s41598-021-84756-y (PMC7970859; doi:10.1038/s41598-021-84756-y)
Supplement: Supplementary file 1 — Supplementary Information 1. [file 41598_2021_84756_MOESM1_ESM.pdf]

POTENZA banda.sav

	hy pn	b1	b2	b3	b4
1	h	2386,057178	2577,284343	2575,822339	2425,367686
2	h	2205,061059	2330,199687	2399,794728	2437,742989
3	h	4677,415769	4797,159684	4941,306115	4973,478896
4	h	1704,849274	1564,380416	1602,510997	1491,473248
5	h	2387,560316	2419,941392	2114,289608	2201,797710
6	h	3150,451203	3373,043008	3427,999511	3621,207304
7	h	3407,670364	3528,282169	3113,139400	3048,334726
8	h	5221,027093	4971,239136	4968,055519	4915,822348
9	h	1885,810615	1963,585725	1958,349784	1855,624659
10	h	2755,178104	2699,399680	2552,206373	2674,497890
11	h	2364,762653	2241,853257	2305,353743	2421,167276
12	h	3780,810485	3766,412937	3487,096198	3694,134241
13	h	4679,265691	4376,572728	4579,011650	4756,778565
14	h	7124,301802	7805,645419	7514,039619	7332,091114
15	h	3084,266565	3087,656580	3057,207939	3187,529851
16	l	2760,974574	3000,382325	3445,176121	2776,429714
17	l	3924,098889	4015,692578	4007,442218	4007,885309
18	l	2551,827324	2799,186172	2646,368820	2789,962870
19	l	3514,089373	3034,838085	2861,457035	3011,156384
20	l	3186,800429	3253,424751	2995,469788	3110,168006
21	l	1680,632373	1969,257887	1653,001939	1761,053565
22	l	2341,753470	2357,204507	2127,350135	2204,863695
23	l	5670,552791	5886,544168	5984,565109	5982,394282
24	l	3158,274747	3282,011657	3451,632840	3402,926257
25	l	3210,566077	3425,419528	3147,164007	2601,037505
26	l	4353,020943	4099,771243	4224,985887	4520,880988
27	l	3657,583806	3881,928043	3803,339629	3965,083564
28	l	4235,065958	4318,842114	4400,652537	4453,859090
29	l	6378,849895	6343,474710	6381,499870	6550,933837
30	l	4111,617833	4182,647456	3649,251828	3655,458378
31	m	4390,031030	4363,761662	4378,807958	4530,454820
32	m	2922,083364	2971,090499	2982,142355	2755,877852
33	m	3125,370895	3153,587648	2856,586633	2952,737444
34	m	2580,334005	2901,024710	2898,557234	2902,223369
35	m	3882,788455	3636,472218	4383,871300	4270,040783
36	m	2728,364067	2420,980294	2390,734479	2386,162248
37	m	2613,257516	2815,929306	2977,697783	2875,852874
38	m	2782,842556	2858,071622	2708,098686	2798,696421

	b5	b6	ss	shss
1	2546,771172	2153,137343	3	10
2	2259,815302	2346,397065	8	8
3	4917,969978	5000,485570	10	8
4	1355,876293	1320,929654	11	11
5	2094,225059	2392,393998	29	8
6	4664,776006	3874,188611	31	10
7	2881,474451	2983,872887	43	9
8	4562,251275	4537,117209	45	8
9	1890,458884	1913,843129	48	11
10	2570,953701	2729,237408	50	9
11	2049,818741	1978,729462	51	11
12	3633,848997	3671,371103	53	11
13	5053,695580	5024,447524	56	8
14	7472,432341	7179,137226	60	12
15	3179,758076	3040,332677	62	10
16	2898,401637	2805,880855	1	0
17	3997,905741	3999,424964	2	0
18	2772,243880	2734,411792	5	2
19	3252,369706	2679,446826	6	1
20	3456,827795	3119,767747	12	2
21	1457,793056	1264,112738	13	2
22	2326,424264	2195,563391	14	1
23	5991,810179	5957,412916	16	0
24	3404,078609	3499,152013	24	1
25	2634,012837	2439,763370	25	4
26	4030,391445	3895,268704	28	0
27	3630,982814	3704,913924	30	0
28	4443,926688	4312,273782	38	0
29	6325,365051	6043,775374	41	1
30	3595,957157	3795,646517	44	2
31	4386,487825	4427,378651	9	6
32	2767,080321	2691,133605	15	7
33	2978,190762	3027,006070	17	6
34	3113,984093	3176,668941	19	5
35	4095,632621	3635,815283	21	5
36	2262,806751	2283,460040	27	7
37	3040,296871	2772,425373	32	7
38	2772,338220	2558,975013	40	5

	mediapotenzabanda
1	2444,07
2	2329,84
3	4884,64
4	1506,67
5	2268,37
6	3685,28
7	3160,46
8	4862,59
9	1911,28
10	2663,58
11	2226,95
12	3672,28
13	4744,96
14	7404,61
15	3106,13
16	2947,87
17	3992,07
18	2715,67
19	3058,89
20	3187,08
21	1630,98
22	2258,86
23	5912,21
24	3366,35
25	2909,66
26	4187,39
27	3773,97
28	4360,77
29	6337,32
30	3831,76
31	4412,82
32	2848,23
33	3015,58
34	2928,80
35	3984,10
36	2412,08
37	2849,24
38	2746,50

POTENZA banda.sav

	hy pn	b1	b2	b3	b4
39	m	3405,447742	3452,092594	3489,442933	3493,565081
40	m	4108,395884	4299,851478	4222,001441	4157,519875
41	m	2802,616791	2672,003009	2697,376042	2510,730527

	b5	b6	ss	shss
39	3492,954303	3375,711403	46	5
40	4183,777276	4104,297826	55	6
41	2505,816017	2617,262961	58	7

	mediapotenzabanda
39	3451,54
40	4179,31
41	2634,30
