# Supplementary material for: Linear and non linear measures of pupil size as a function of hypnotizability
Source: Sci Rep. 2021 Mar 4;11:5196. doi: 10.1038/s41598-021-84756-y (PMC7970859; doi:10.1038/s41598-021-84756-y)
Supplement: Supplementary file 2 — Supplementary Information 2. [file 41598_2021_84756_MOESM2_ESM.pdf]

frequenza mediana.sav

	hypn	VAR00001	b1	b2	b3
1	h	1,00	,015347459	,008337795	,008334803
2	h	1,00	,015338867	,008340821	,008335775
3	h	1,00	,015339568	,008338611	,008338332
4	h	1,00	,015340053	,008338003	,008333949
5	h	1,00	,015368896	,008352505	,008351782
6	h	1,00	,015361923	,008439092	,008389653
7	h	1,00	,015360419	,008361491	,008394847
8	h	1,00	,015335021	,008335238	,008335872
9	h	1,00	,015363567	,008354663	,008347591
10	h	1,00	,015336925	,008338681	,008336096
11	h	1,00	,015402765	,008421289	,008537621
12	h	1,00	,015343126	,008363895	,008362240
13	h	1,00	,015336807	,008337786	,008335816
14	h	1,00	,015361013	,008339680	,008350548
15	h	1,00	,015339124	,008332578	,008340715
16	l	3,00	,015401671	,008388271	,008354148
17	l	3,00	,015334220	,008332562	,008333158
18	l	3,00	,015360575	,008339748	,008370326
19	l	3,00	,015373295	,008371495	,008360053
20	l	3,00	,015345156	,008345505	,008347166
21	l	3,00	,015494406	,008348604	,008360179
22	l	3,00	,015345540	,008349064	,008374854
23	l	3,00	,015334643	,008335870	,008336964
24	l	3,00	,015333474	,008334920	,008338042
25	l	3,00	,015369361	,008341249	,008349925
26	l	3,00	,015342326	,008345949	,008341867
27	l	3,00	,015344703	,008333797	,008336688
28	l	3,00	,015338445	,008336440	,008336709
29	l	3,00	,015333936	,008333929	,008334135
30	l	3,00	,015342790	,008347045	,008356998
31	m	2,00	,016044909	,008336149	,008333702
32	m	2,00	,016047063	,008338864	,008335656
33	m	2,00	,016064739	,008340226	,008444658
34	m	2,00	,016091353	,008359642	,008366090
35	m	2,00	,016140726	,008373522	,008358188
36	m	2,00	,016045898	,008338160	,008336144
37	m	2,00	,016072541	,008346978	,008371907
38	m	2,00	,016094006	,008376788	,008366289
39	m	2,00	,016039608	,008333904	,008335438

	b4	b5	b6
1	,008352492	,008339725	,008334127
2	,008339555	,008348121	,008337154
3	,008341166	,008341862	,008335587
4	,008336452	,008338001	,008350949
5	,008371147	,008352320	,008355741
6	,008424556	,008491922	,008406270
7	,008345323	,008371447	,008350198
8	,008338299	,008340530	,008333888
9	,008347326	,008342812	,008348128
10	,008335430	,008340947	,008349364
11	,008560702	,008488070	,008434702
12	,008352993	,008362966	,008362460
13	,008338001	,008334552	,008335642
14	,008346184	,008339971	,008341432
15	,008333215	,008336068	,008341977
16	,008352686	,008360108	,008361230
17	,008332886	,008333994	,008333778
18	,008349886	,008358531	,008356047
19	,008375521	,008371237	,008380929
20	,008343119	,008354391	,008361193
21	,008408130	,008371248	,008410109
22	,008339756	,008344860	,008340163
23	,008340352	,008338851	,008343811
24	,008336982	,008335244	,008336057
25	,008347531	,008351951	,008341079
26	,008365699	,008351652	,008341177
27	,008336686	,008339770	,008337517
28	,008337506	,008335516	,008338817
29	,008334720	,008336933	,008337698
30	,008350661	,008355041	,008351957
31	,008333404	,008336456	,008334711
32	,008336555	,008334403	,008334010
33	,008350563	,008347117	,008368223
34	,008391251	,008341520	,008361270
35	,008360577	,008350021	,008359972
36	,008342136	,008343496	,008345302
37	,008368612	,008378531	,008363118
38	,008360224	,008356072	,008357883
39	,008334000	,008336920	,008334549

	hypn	VAR00001	b1	b2	b3
40	m	2,00	,016044846	,008334632	,008334211
41	?	?	?	?	?
42	?	?	?	?	?

	b4	b5	b6
40	,008335935	,008335947	,008342164
41	,008353845	,008348197	,008351092
42	.	.	.
