# Supplementary material for: Linear and non linear measures of pupil size as a function of hypnotizability
Source: Sci Rep. 2021 Mar 4;11:5196. doi: 10.1038/s41598-021-84756-y (PMC7970859; doi:10.1038/s41598-021-84756-y)
Supplement: Supplementary file 3 — Supplementary Information 3. [file 41598_2021_84756_MOESM3_ESM.pdf]

determinism.sav

	hy pn	b1	b2	b3	b4
1	h	,998563695	,999382760	,998722542	,999863955
2	h	,996185703	,999133464	,996388664	,999348226
3	h	,998462848	,998847096	,999145442	,999014379
4	h	,996925850	,997124818	,994996123	,998359594
5	h	,999773953	,999930787	,999724414	,999850081
6	h	,999782760	,999999835	,999980111	,999998838
7	h	,999884876	,999956525	,999987535	,999849088
8	h	,999019479	,999235076	,998958579	,999898583
9	h	,999528093	,999926226	,999419793	,998568363
10	h	,996083104	,999213805	,998723249	,998138391
11	h	,999949382	,999970829	,999999837	,999999838
12	h	,999744174	,999962579	,999812083	,999745652
13	h	,998262994	,999651077	,998966647	,999698740
14	h	,999994163	,999818921	,999963348	,999928951
15	h	,998854982	,996918179	,999867025	,995811753
16	l	,999900245	,999945262	,999878430	,999918845
17	l	,995943609	,992140784	,994120830	,994808523
18	l	,999847368	,999451143	,999967180	,999912662
19	l	,999981982	,999994236	,999964877	,999967007
20	l	,999724067	,999659160	,999795003	,999190821
21	l	,999926141	,999845903	,999891657	,999938045
22	l	,999368388	,999898519	,999958915	,999616169
23	l	,999045633	,998092432	,995875625	,998568594
24	l	,998206342	,999302855	,999725488	,999820797
25	l	,999911197	,999722980	,999864309	,999828800
26	l	,998922750	,999531959	,999761182	,999979845
27	l	,999624276	,996875993	,999199173	,999506941
28	l	,999389526	,998904850	,999540686	,999060207
29	l	,998338532	,998786508	,998748949	,999229632
30	l	,998341045	,999733847	,999953737	,999848282
31	m	,999074201	,999136319	,996984040	,996711622
32	m	,998755558	,999081765	,996814259	,999574176
33	m	,999848657	,999736453	,999999836	,999807836
34	m	,999971031	,999975775	,999981271	,999990255
35	m	,999997515	,999989311	,999649623	,999950356
36	m	,999505365	,998847676	,997781203	,999173319
37	m	,999927332	,999975412	,999928299	,999981110
38	m	,999730739	,999864922	,999863102	,999666960

	b5	b6	media
1	,999612402	,999011448	1,00
2	,999814381	,997723458	1,00
3	,999333182	,999220577	1,00
4	,996816356	,999281475	1,00
5	,999579919	,999852447	1,00
6	,999999842	,999999833	1,00
7	,999986690	,999940067	1,00
8	,999771165	,998201033	1,00
9	,999439693	,999774752	1,00
10	,999524343	,999816477	1,00
11	,999999831	,999983686	1,00
12	,999855713	,999930906	1,00
13	,997899299	,999021520	1,00
14	,999839590	,999919911	1,00
15	,999108326	,999599683	1,00
16	,999875204	,999904742	1,00
17	,996867325	,996012452	,99
18	,999942593	,999971203	1,00
19	,999960176	,999934067	1,00
20	,999824528	,999926534	1,00
21	,999777729	,999796808	1,00
22	,999842446	,999775067	1,00
23	,997846262	,998195183	1,00
24	,999571889	,999766972	1,00
25	,999796668	,999816378	1,00
26	,999966113	,999281183	1,00
27	,999728881	,999657868	1,00
28	,997223244	,999135291	1,00
29	,999852098	,999627088	1,00
30	,999912118	,999736063	1,00
31	,998767833	,997666062	1,00
32	,995275408	,997115523	1,00
33	,999682770	,999912968	1,00
34	,999759625	,999972014	1,00
35	,999617527	,999907809	1,00
36	,999047956	,999591179	1,00
37	,999980421	,999960557	1,00
38	,999703753	,999640946	1,00

	hy pn	b1	b2	b3	b4
39	m	,998419171	,999178414	,998624719	,998035723
40	m	,999687932	,998849980	,998399971	,999679643
41	m	,999653239	,999977038	,999969013	,999874732

	b5	b6	media
39	,999725479	,998217748	1,00
40	,999401544	,999796815	1,00
41	,999729541	,999771128	1,00
