# Supplementary material for: Linear and non linear measures of pupil size as a function of hypnotizability
Source: Sci Rep. 2021 Mar 4;11:5196. doi: 10.1038/s41598-021-84756-y (PMC7970859; doi:10.1038/s41598-021-84756-y)
Supplement: Supplementary file 4 — Supplementary Information 4. [file 41598_2021_84756_MOESM4_ESM.pdf]

	hy pn	b1	b2	b3	b4
1	h	4,321101494	4,712836292	4,592662083	5,560020024
2	h	3,746307827	4,644117153	3,737177897	4,919347535
3	h	4,340398422	4,376957205	4,698206863	4,440519532
4	h	4,156233709	4,080453950	3,900684955	4,552577668
5	h	5,316576853	6,228879401	5,634356094	5,343456632
6	h	5,461118820	8,155074888	6,589101805	7,058986343
7	h	5,916039865	5,991238959	6,649957619	5,962694969
8	h	4,916630647	4,620054170	4,483005999	5,793889619
9	h	4,874068776	5,799963664	4,651938687	4,324980751
10	h	3,794325806	4,808365713	4,713956253	4,333790484
11	h	5,972771763	6,447653646	8,159660737	8,163086376
12	h	5,429151295	6,064326100	5,280487870	5,031821931
13	h	4,498311912	5,319784726	4,799884018	5,316775399
14	h	6,792417598	5,532967105	6,641502037	6,246593790
15	h	4,163887749	4,045075736	5,194953440	3,877851382
16	l	5,579437229	5,758684993	5,694030113	5,791120447
17	l	4,004526815	3,679765362	3,740559388	3,962836090
18	l	5,488879357	5,019353349	6,132002726	5,652087899
19	l	6,623772049	6,019273322	6,342055307	6,204133456
20	l	5,339770559	4,952725462	5,150329312	4,509559080
21	l	5,856159599	5,615108808	6,024895803	6,193197351
22	l	4,839700071	5,637772603	6,221608638	5,356731719
23	l	5,053095601	4,263954795	3,812811953	4,138886426
24	l	4,361500957	4,978632497	5,085917717	5,743199579
25	l	5,795790516	5,175726172	5,405295633	5,349761823
26	l	4,359555400	4,826023933	5,087048684	5,616555888
27	l	5,097197048	4,017242344	4,754782632	4,971950330
28	l	4,759069986	4,528616101	4,764343855	4,682443264
29	l	4,817177728	4,880322549	4,991349416	5,234573622
30	l	4,454433815	5,040361196	6,325852927	5,415598605
31	m	4,602518935	4,709106005	4,204883868	4,082432413
32	m	4,675365423	4,645269222	3,637496572	4,835182642
33	m	5,631360774	5,506282312	8,158802491	5,328243149
34	m	5,956463193	6,623804186	6,613347436	7,127300137
35	m	6,588989019	6,596750528	4,939222893	5,715324997
36	m	5,199480071	4,604366614	4,274702338	4,571917781
37	m	5,763380398	6,640911855	5,807338104	6,533964658
38	m	5,267272726	5,064191219	5,240399213	5,024616111

	b5	b6	media
1	4,865764440	5,018402216	4,85
2	5,618519984	4,187550565	4,48
3	4,689443578	4,879470578	4,57
4	3,868808516	4,731416280	4,22
5	4,942185710	5,379775203	5,47
6	8,176672772	8,150179270	7,27
7	6,880357149	6,633172406	6,34
8	5,287951210	4,150087468	4,88
9	4,873693336	5,262900736	4,96
10	5,058671475	5,751999179	4,74
11	8,144098463	6,530524370	7,24
12	5,497269652	5,907297753	5,54
13	4,644422825	4,837653350	4,90
14	5,755507900	5,991236488	6,16
15	4,591191142	5,178720927	4,51
16	5,327397102	5,573221289	5,62
17	4,458909833	4,109553507	3,99
18	5,848168076	6,266874949	5,73
19	6,125039897	6,093520812	6,23
20	5,391432180	5,798168233	5,19
21	5,608385526	5,451082848	5,79
22	5,567209860	5,194155262	5,47
23	4,225213749	3,910917108	4,23
24	5,226268396	5,570457577	5,16
25	5,511877654	5,725588486	5,49
26	5,871526214	4,670091035	5,07
27	5,355397406	5,170686891	4,89
28	4,044611972	4,623931189	4,57
29	6,055573034	5,492661803	5,25
30	5,812795294	4,985512397	5,34
31	4,695835824	4,150507034	4,41
32	3,710059975	4,083589570	4,26
33	4,831601508	5,718068271	5,86
34	5,413534578	6,404660607	6,36
35	5,010577792	5,807069921	5,78
36	4,539444828	5,062654180	4,71
37	5,947475050	6,251336463	6,16
38	5,424174374	5,005096820	5,17

	hypn	b1	b2	b3	b4
39	m	4,403052430	4,768356313	4,371654281	4,188771100
40	m	5,108749546	4,783851580	4,384045923	5,128846955
41	?	?	?	?	?

	b5	b6	media
39	5,592827459	4,155957568	4,58
40	5,121206003	5,333485442	4,98
41	5,454597885	5,022325390	5,73
