# Supplementary material for: Linear and non linear measures of pupil size as a function of hypnotizability
Source: Sci Rep. 2021 Mar 4;11:5196. doi: 10.1038/s41598-021-84756-y (PMC7970859; doi:10.1038/s41598-021-84756-y)
Supplement: Supplementary file 5 — Supplementary Information 5. [file 41598_2021_84756_MOESM5_ESM.pdf]

	hypn	ss	b1	b2	b3
1	h	3	1,139348221	1,041560936	,740464115
2	h	8	,889915276	1,005428653	,781823925
3	h	10	1,202207756	1,233053780	1,160899925
4	h	11	,632666492	,766611099	,535509729
5	h	29	1,542633269	,893625927	1,558554840
6	h	31	1,397448540	2,745468235	2,167428398
7	h	43	1,561581898	2,941780949	3,915290737
8	h	45	,768595791	1,319012451	1,312828445
9	h	48	1,072202015	,979326659	1,047089367
10	h	50	,860317802	,913334973	,804489279
11	h	51	1,244243622	2,204367924	2,183256107
12	h	53	,639799976	1,770859039	2,145113087
13	h	56	,908223152	1,086491340	1,022858429
14	h	60	1,262830353	2,048413467	2,191077805
15	h	62	,636556244	,532729244	,879603958
16	l	1	2,636418533	3,356521320	2,019361639
17	l	2	,790745219	,673500443	,701500851
18	l	5	1,592520237	1,317304230	1,806576633
19	l	6	1,086463737	1,795899868	1,440250015
20	l	12	1,039283085	1,424501324	1,341756891
21	l	13	1,031587696	1,186729622	1,405560780
22	l	14	,857250023	1,388332939	3,055978680
23	l	16	,819780159	,945336533	1,004238319
24	l	24	,630694771	,616094589	1,126461697
25	l	25	1,233289242	,877977943	2,198011780
26	l	28	,789353561	1,489543438	1,149552727
27	l	30	1,129536057	,787532884	1,173537827
28	l	38	,891922705	1,107545840	,800124950
29	l	41	,811596489	,996389008	,919333267
30	l	44	1,278680897	1,979275227	1,590260601
31	m	9	1,334491253	,909432507	,803403888
32	m	15	,950306320	1,176695156	1,008371830
33	m	17	,677642250	,741872787	4,644282640
34	m	19	2,295569038	1,436588669	1,421398926
35	m	21	3,345760515	2,024350166	2,843329620
36	m	27	,845133400	,741658211	,762565899
37	m	32	2,502471066	1,266564751	2,834889126
38	m	40	1,938937283	2,795925585	1,874125862

MAD.sav

	b4	b5	b6	shss	mediaMAD
1	1,770652771	1,295753384	,427817535	10	1,07
2	,795581341	1,521715927	,833229139	8	,97
3	1,272131729	1,285608482	,724798584	8	1,15
4	,521420097	,813437843	1,064347124	11	,72
5	1,727384333	1,496608448	1,861847305	8	1,51
6	4,366335773	3,554306221	2,542554665	10	2,80
7	1,642781734	2,331840420	1,498307037	9	2,32
8	1,209395599	1,680320549	,790414238	8	1,18
9	1,176697479	,956325531	1,233845291	11	1,08
10	,824217701	1,327659988	1,958872890	9	1,11
11	3,900791954	2,649931526	2,223015308	11	2,40
12	1,612440335	1,876513418	1,904007526	11	1,66
13	1,171764755	,762359428	1,079778753	8	1,01
14	1,691838837	2,247727013	1,877685165	12	1,89
15	,619039440	,907388496	,861913300	10	,74
16	1,183520317	1,854049635	2,087876415	0	2,19
17	,686545410	,840372467	,908108071	0	,77
18	1,398260117	2,320376396	1,929609394	2	1,73
19	2,437481308	2,504478931	2,816745380	1	2,01
20	1,569749451	1,904358768	2,001077938	2	1,55
21	2,871872616	1,904505253	2,348843375	2	1,79
22	,896773720	1,301659203	,775499630	1	1,38
23	2,018047142	1,493383217	1,898563508	0	1,36
24	,829859352	,679314613	,677454567	1	,76
25	1,700646400	2,083412933	,955127907	4	1,51
26	2,649996758	1,963132524	1,494318008	0	1,59
27	1,057775497	1,184533215	1,012017441	0	1,06
28	1,076985529	1,002868462	1,239827919	0	1,02
29	,958343887	1,544565570	1,532490730	1	1,13
30	1,764833736	2,164311600	2,293506227	2	1,85
31	,661358643	1,116267776	,947691917	6	,96
32	,578946781	,674073792	,735218525	7	,85
33	1,498351479	1,026826000	1,598877811	6	1,70
34	3,325407505	1,305342484	2,110848808	5	1,98
35	2,427916145	1,973353767	2,026947403	5	2,44
36	1,406983471	1,373839378	1,385535812	7	1,09
37	2,219246483	3,672499752	2,349411774	7	2,47
38	1,917988968	2,016764259	1,873111534	5	2,07

	hy pn	ss	b1	b2	b3
39	m	46	,851740789	,621929640	,967450714
40	m	55	1,209571457	,720603180	,872462940
41	m	58	1,541669369	2,151872968	1,345548344

	b4	b5	b6	shss	mediaMAD
39	,837979507	,906857967	,803356552	5	,83
40	1,116003227	,930106068	1,202517509	6	1,01
41	1,519321537	1,319845581	1,596358681	7	1,58
