# Supplementary material for: Linear and non linear measures of pupil size as a function of hypnotizability
Source: Sci Rep. 2021 Mar 4;11:5196. doi: 10.1038/s41598-021-84756-y (PMC7970859; doi:10.1038/s41598-021-84756-y)
Supplement: Supplementary file 6 — Supplementary Information 6. [file 41598_2021_84756_MOESM6_ESM.pdf]

	hypn	b1	b2	b3	b4
1	h	48,81883265	50,74632813	50,74103770	49,18467798
2	h	46,94386916	48,24377032	48,97372421	49,34823024
3	h	68,36937950	69,22999252	70,26359135	70,47998877
4	h	41,27573189	39,53568210	40,02433850	38,60701052
5	h	48,80013848	49,12958686	45,92421447	46,81071534
6	h	56,06977363	57,70482802	58,34416667	59,84146990
7	h	58,31663653	59,29101156	55,58299518	55,16438955
8	h	72,24387084	70,48916863	70,46391450	70,08231383
9	h	43,37786956	44,24961978	44,20929791	43,03486573
10	h	52,47723073	51,93187143	50,50398073	51,70186199
11	h	48,51316961	47,09376030	47,42959590	48,54078305
12	h	61,46115926	61,25037505	58,94130218	60,69939245
13	h	68,38905332	66,12882124	67,64892329	68,94052307
14	h	84,31919284	88,30367596	86,58214040	85,54980452
15	h	55,51890541	55,56148654	55,25987619	56,45077524
16	lows	52,42186912	54,58849602	58,61431669	52,62345037
17	lows	62,63316208	63,36362720	63,29623652	63,30076948
18	lows	50,46463342	52,87964210	51,32193933	52,76039090
19	lows	59,19542455	54,95602663	53,39962143	54,72802971
20	lows	56,42310677	56,98927687	54,67792203	55,72842375
21	lows	40,77689112	44,32956007	40,58615958	41,77203497
22	lows	48,36650608	48,49855539	46,00234029	46,93137469
23	lows	75,29065553	76,70149219	77,33238541	77,30265041
24	lows	56,19140390	57,27543620	58,72585110	58,31374061
25	lows	56,58849149	58,49118294	56,03604422	50,94990249
26	lows	65,95002778	63,97216129	64,95761369	67,09798564
27	lows	60,44816459	62,29473707	61,65024948	62,94750350
28	lows	65,05858908	65,69652866	66,31477155	66,71127161
29	lows	79,85637381	79,63203003	79,86935672	80,91986984
30	lows	64,09430106	64,61127498	60,31506941	60,38923931
31	m	66,23520723	66,03844707	66,16190808	67,29902069
32	m	54,03463996	54,48206488	54,59380575	52,47902365
33	m	55,85186404	56,12580960	53,08621231	54,27547179
34	m	50,70670722	53,76887442	53,72528786	53,67859471
35	m	62,10607333	60,14992984	66,10311168	65,22993773
36	m	52,21471186	49,18238527	48,88010494	48,81580218
37	m	51,05903120	53,01457782	54,43486712	53,50639375
38	m	52,65455790	53,31468677	51,92958945	52,81022458
39	m	58,34638700	58,74434028	59,05584557	59,09581488

	b5	b6	ss	shss	media
1	50,43919798	46,39325873	3	10	49,39
2	47,48879685	48,42179846	8	8	48,24
3	70,08264652	70,69473413	10	8	69,85
4	36,80680546	36,30120028	11	11	38,76
5	45,70431134	48,83969372	29	8	47,53
6	67,64901494	61,96376174	31	10	60,26
7	53,54959338	54,56207043	43	9	56,08
8	67,50591918	67,34653252	45	8	69,69
9	43,44866596	43,70264040	48	11	43,67
10	50,67438696	52,18468095	50	9	51,58
11	44,85411920	44,20866499	51	11	46,77
12	60,16616735	60,47782904	53	11	60,50
13	71,07428194	70,86367725	56	8	68,84
14	86,39682471	84,67688745	60	12	85,97
15	56,37226583	55,10298598	62	10	55,71
16	53,74306191	52,87478215	1	0	54,14
17	63,21770760	63,23053802	2	0	63,17
18	52,56538004	52,21323276	5	2	52,03
19	56,89239450	51,60901346	6	1	55,13
20	58,71249407	55,75400614	12	2	56,38
21	38,08920481	35,38678907	13	2	40,16
22	48,19300428	46,83114618	14	1	47,47
23	77,37042369	77,12508897	16	0	76,85
24	58,32969270	59,13575502	24	1	58,00
25	51,25828140	49,36416484	25	4	53,78
26	63,40689400	62,37407506	28	0	64,63
27	60,22601303	60,84428203	30	0	61,40
28	66,64479888	65,63719492	38	0	66,01
29	79,50394958	77,71057726	41	1	79,58
30	59,88003160	61,53154503	44	2	61,80
31	66,20896666	66,52381005	9	6	66,41
32	52,59236411	51,86682895	15	7	53,34
33	54,52015274	54,89579484	17	6	54,79
34	55,76794807	56,25989451	19	5	53,98
35	63,92426863	60,19321892	21	5	62,95
36	47,53338835	47,74465277	27	7	49,06
37	54,98232698	52,55268105	32	7	53,26
38	52,57400700	50,50494954	40	5	52,30
39	59,08030270	58,08856847	46	5	58,74

	hypn	b1	b2	b3	b4
40	m	64,07551346	65,55903815	64,96448827	64,45981962
41	?	?	?	?	?

	b5	b6	ss	shss	media
40	64,66300478	64,02198435	55	6	64,62
41	50,00659957	51,09767203	58	7	51,22
