# Supplementary material for: Linear and non linear measures of pupil size as a function of hypnotizability
Source: Sci Rep. 2021 Mar 4;11:5196. doi: 10.1038/s41598-021-84756-y (PMC7970859; doi:10.1038/s41598-021-84756-y)
Supplement: Supplementary file 7 — Supplementary Information 7. [file 41598_2021_84756_MOESM7_ESM.pdf]

	hy pn	b1	b2	b3	b4
1	h	49,01966038	50,82827053	50,74323187	48,85901222
2	h	46,97207193	48,47395625	49,12086344	49,30011139
3	h	68,86376343	69,83295364	70,90230312	71,16887054
4	h	41,38728342	39,80420923	39,94344749	38,56969395
5	h	48,95930683	49,35733376	45,91258011	47,03800802
6	h	56,89242449	57,21725712	59,26716585	59,00838852
7	h	58,71574945	60,10852165	56,44632406	55,45869513
8	h	72,33828220	70,63761177	70,56388035	69,97369480
9	h	43,66105671	44,57776931	44,20030039	43,10166111
10	h	52,48025579	51,85481437	50,48181291	51,66603537
11	h	49,20540323	46,75507822	44,66113514	46,47192471
12	h	61,78269730	61,67363720	59,06271591	60,66781139
13	h	68,32809353	66,57957311	67,36657867	69,52910576
14	h	85,72575493	88,64376431	88,03315659	87,06339912
15	h	55,84047260	55,49634104	55,92300339	56,54321337
16	l	52,83860188	55,47110100	59,20033746	52,70244093
17	l	62,54180760	63,49013624	63,39061226	63,33914661
18	l	50,90929098	52,69975786	50,74621229	52,56096125
19	l	59,92284517	56,35529966	54,08453875	55,14907751
20	l	57,12087660	56,82259283	54,88644533	56,09326515
21	l	39,26342983	44,78312473	40,84904680	41,03695078
22	l	48,65933514	49,07722454	46,07627058	47,19839640
23	l	75,81710033	77,32636280	78,14432793	77,76700592
24	l	56,30107222	57,67906027	59,13421211	58,55060692
25	l	57,75571833	58,50444250	55,95356264	51,25362806
26	l	66,16295261	64,38049364	64,87651625	68,72225838
27	l	60,69252310	62,35894308	61,62558804	62,60537024
28	l	65,38708959	66,15224829	66,57974203	67,12326715
29	l	80,13788433	79,96941299	80,25749836	81,33191662
30	l	64,56559973	65,02884169	60,87042398	61,00750370
31	m	66,04737396	66,33530788	66,22552473	67,56105232
32	m	54,36678486	54,57719030	54,79087591	52,65887938
33	m	56,59250536	56,59554462	51,73425451	53,90198030
34	m	50,90892735	54,56067305	53,21096067	52,98291531
35	m	61,88985748	61,71627893	66,50014076	66,41219501
36	m	52,72041950	49,53830938	48,92755508	49,00387669
37	m	51,49263973	53,36159458	54,28352451	54,50423307
38	m	52,67968178	54,01255391	52,73413467	53,73850822

	b5	b6	ss	shss	mediabasale
1	50,70690756	46,34459944	3	10	49,42
2	47,42489977	48,57568596	8	8	48,31
3	70,85514355	71,07280025	10	8	70,45
4	36,92942591	36,42614183	11	11	38,84
5	45,64686680	48,95982161	29	8	47,65
6	69,61140385	61,95246744	31	10	60,66
7	53,27819653	54,43657360	43	9	56,41
8	66,85485249	67,42071819	45	8	69,63
9	43,52425022	43,55213591	48	11	43,77
10	50,72767611	52,34002342	50	9	51,59
11	43,58109999	44,43387012	51	11	45,85
12	60,96349295	61,26511126	53	11	60,90
13	71,18763084	70,89042195	56	8	68,98
14	86,23991375	84,71970215	60	12	86,74
15	56,18650026	55,43380280	62	10	55,90
16	54,08203864	52,84222536	1	0	54,52
17	63,12995338	63,36479607	2	0	63,21
18	52,38836927	52,31494160	5	2	51,94
19	57,82552357	51,51111631	6	1	55,81
20	58,63341379	55,26938982	12	2	56,47
21	37,91371155	34,69079486	13	2	39,76
22	48,62094069	46,90049191	14	1	47,76
23	77,92211151	78,22687270	16	0	77,53
24	58,81392908	59,65190172	24	1	58,36
25	51,28588629	49,09902382	25	4	53,98
26	63,33160734	62,20081139	28	0	64,95
27	60,42937403	61,07143373	30	0	61,46
28	66,74772034	66,08748264	38	0	66,35
29	79,65105878	77,95052853	41	1	79,88
30	60,57239666	61,65033007	44	2	62,28
31	66,27206326	66,67407894	9	6	66,52
32	52,72800074	51,94271994	15	7	53,51
33	54,58836727	53,94378014	17	6	54,56
34	55,94017324	56,33038778	19	5	53,99
35	64,01397285	60,29736252	21	5	63,47
36	47,47947464	48,03651447	27	7	49,28
37	55,37687912	52,73005266	32	7	53,62
38	53,12860537	50,63007917	40	5	52,82

	hy pn	b1	b2	b3	b4
39	m	58,33203192	58,90308225	59,03785934	59,08583059
40	m	64,48961716	65,94704704	65,03655396	64,69366417
41	m	52,91392536	52,45439844	51,74795780	50,41603727

mediana.sav

	b5	b6	ss	shss	mediabasale
39	59,35534840	58,27472200	46	5	58,83
40	64,76338720	63,71504288	55	6	64,77
41	50,61665134	50,97504168	58	7	51,52
