# Supplementary material for: Linear and non linear measures of pupil size as a function of hypnotizability
Source: Sci Rep. 2021 Mar 4;11:5196. doi: 10.1038/s41598-021-84756-y (PMC7970859; doi:10.1038/s41598-021-84756-y)
Supplement: Supplementary file 8 — Supplementary Information 8. [file 41598_2021_84756_MOESM8_ESM.pdf]

	hypn	ss	b1	b2	b3
1	h	3	1,667386538	1,447448374	1,081553125
2	h	8	1,155374120	1,655014455	1,170233009
3	h	10	1,745070732	2,090223165	2,082077980
4	h	11	1,078807332	1,144823624	,750654058
5	h	29	2,471825694	2,495358845	2,292944245
6	h	31	2,575864615	6,573266277	4,895342846
7	h	43	2,616058937	3,586319660	4,865862886
8	h	45	1,360575521	1,586486862	1,700904273
9	h	48	2,042836308	2,357629773	1,972014947
10	h	50	1,148491672	1,575150613	1,246890407
11	h	51	3,352725909	4,902822931	7,470118613
12	h	53	1,827044756	3,848195054	3,608699398
13	h	56	1,484656128	1,884864918	1,623440740
14	h	60	3,818765816	2,847539841	4,192548609
15	h	62	1,385167087	,760232396	1,885473539
16	l	1	3,595666614	4,525938010	3,088794150
17	l	2	1,089267005	,862283278	1,014369916
18	l	5	2,269521804	1,711852012	3,525736256
19	l	6	3,161675708	3,831096168	3,152812392
20	l	12	1,798639589	2,376686996	2,407538059
21	l	13	4,229262660	2,036944560	2,401497732
22	l	14	1,560706265	2,257442727	3,337351656
23	l	16	1,367822068	1,851003892	2,066025302
24	l	24	,895144637	1,239552867	1,705303343
25	l	25	2,883219659	2,049930008	2,669781854
26	l	28	1,901743038	2,708479310	2,344320259
27	l	30	1,898695777	1,137601207	1,608442434
28	l	38	1,564351632	1,676012034	1,733333713
29	l	41	1,345504348	1,488327271	1,544794209
30	l	44	1,881545572	2,834225192	3,368587344
31	m	9	1,711700680	1,638876753	1,187547589
32	m	15	1,530457742	1,672089239	1,288095087
33	m	17	2,437899572	1,866041782	6,200917125
34	m	19	3,027994123	3,152080658	3,486266395
35	m	21	5,063379627	4,296892547	3,775431758
36	m	27	1,410317697	1,440086441	1,212529489
37	m	32	2,497234951	2,320633384	3,814061501
38	m	40	3,216460425	3,952231451	3,379289382

SD.sav

	b4	b5	b6	shss	media
1	2,497372625	1,630710555	,896164383	10	1,54
2	1,579827330	2,151920508	1,314145737	8	1,50
3	2,460032222	2,528717391	1,655565632	8	2,09
4	,986030795	1,065683192	1,775777950	11	1,13
5	3,249241575	2,311377806	2,660879801	8	2,58
6	6,341683758	9,402730217	5,889862111	10	5,95
7	2,286110977	3,730866632	2,618255325	9	3,28
8	2,071914350	2,281138999	1,249880206	8	1,71
9	1,904205297	1,634947330	1,980767558	11	1,98
10	1,189852603	1,749587735	2,449111655	9	1,56
11	8,060874749	6,159323588	4,932561041	11	5,81
12	3,117803167	3,726279681	3,715794815	11	3,31
13	1,995983735	1,463769528	1,669593970	8	1,69
14	3,650446632	2,832533942	2,994068803	12	3,39
15	,916547132	1,387896245	1,998680222	10	1,39
16	2,684061493	3,176119380	3,184506886	0	3,38
17	,947703255	1,194815325	1,150823194	0	1,04
18	2,511129912	3,021131598	2,862235514	2	2,65
19	4,000448902	3,953415605	3,995120608	1	3,68
20	2,124157536	3,110228496	3,355841802	2	2,53
21	4,019346438	2,647164378	3,448361747	2	3,13
22	1,520001898	1,964605587	1,551711196	1	2,03
23	2,587736675	2,372610544	3,022598824	0	2,21
24	1,560317037	1,313787600	1,454330756	1	1,36
25	2,268561254	2,569680540	1,715638912	4	2,36
26	4,329724873	3,155947494	2,178252046	0	2,77
27	1,641985482	1,952236217	1,699432422	0	1,66
28	1,861798152	1,548591702	2,008368891	0	1,73
29	1,705670118	2,118560584	2,200659200	1	1,73
30	2,932668131	3,215873851	3,085146045	2	2,89
31	1,138856767	1,691553705	1,400675693	6	1,46
32	1,352936213	1,060127431	,982816074	7	1,31
33	2,629168236	2,396936003	3,668994039	6	3,20
34	4,564824862	1,980189416	3,390634768	5	3,27
35	3,885897472	3,053373543	3,548968495	5	3,94
36	1,783420324	1,839750571	1,977184353	7	1,61
37	3,594758752	4,152755629	3,262520927	7	3,27
38	3,127189867	2,883455732	2,868339113	5	3,24

	hy pn	ss	b1	b2	b3
39	m	46	1,07120495[illegible]	1,09334881[illegible]	1,36034957[illegible]
40	m	55	1,65103491[illegible]	1,36547162[illegible]	1,271673794
41	m	58	3,01778218[illegible]	4,50693542[illegible]	3,47140499[illegible]

	b4	b5	b6	shss	media
39	1,118074779	1,572520888	1,195831435	5	1,24
40	1,565953421	1,572824385	2,341979815	6	1,63
41	2,619571097	2,271001791	2,508509824	7	3,07
